# Emotional intelligence: An important skill to learn now more than ever

**DOI:** 10.12688/f1000research.134752.1

**Published:** 2023-09-13

**Authors:** Nikhilesh Anand, Vasavi Rakesh Gorantla, Rajeev Ranjan, Hani Morcos

**Affiliations:** 1 Department of Pharmacology, American University of Antigua College of Medicine, St. John's, 33901, Antigua and Barbuda; 2 Department of Biomedical Sciences, Research Faculty, West Virginia School of Osteopathic Medicine, Lewisburg, West Virginia, 24901, USA; 3 Department of Psychiatry, All India Institute of Medical Sciences, Patna, Bihar, 801507, India

**Keywords:** Emotional Intelligence, Multifactor EI scale, Bar-On Emotional Quotient, Trait EI Questionnaire.

## Abstract

Emotional intelligence (EI) is defined as ability of an individual to perceive, understand, regulate, control and utilise emotions. EI is a relatively nascent concept from the 1900s that attempts to connect emotion to general intelligence. Several clinical studies and survey research have confirmed positive association between EI and optimal mental and physical health, overall well-being, and on-aggressive behaviours. In this review, a brief overview on evolutional theory of EI, various models associated with EI, methods of classifying measures, and comparing various EI measurement methods are described. Indeed, EI assessment measures such as Multifactor EI Scale, Mayer-Salovey-Caruso Tests, Self-report Test, Trait EI Questionnaire, Bar-On Emotional Quotient Inventory, The Situational Test of Emotional Management, The Situational Test of Emotional Understanding, Emotional and Social competence Inventory are also discussed. The key purpose of this review is to encapsulate important components of EI to audiences, reap the benefits of evaluating EI and implying attributes to improve their emotional health and general well-being.

**Abbreviations**
EI: Emotional intelligence
EQ-i: Bar-On Emotional Quotient Inventory
ESCI: Emotional and Social competence Inventory
MEIS: Multifactor EI Scale
MSCEIT: Mayer-Salovey-Caruso EI Tests
SREIT: Self-report EI Test
STEM: Situational Test of Emotional Management
STEU: The Situational Test of Emotional Understanding
TEIQue: Trait EI Questionnaire

## Introduction

Human beings are an intricate species of emotion and cognition. Even though emotions are known to play a crucial role in our lives, their underlying mechanisms are yet to be revealed clearly and little is known about its correlation with cognitive processes. Generally, reasoning ability enables us to judge or rationalize the concepts or constructs, and make decisions, while emotions help us to understand and empathize with others. A traditional view on highly intelligent people has good reasoning skills, profound memory, and a well-defined logical mind, which was supported by intelligent quotient (IQ) tests, designed to determine to test an individuals’ intelligence and capability based only on the logical reasoning and the aptitude, ^1^ is now revised after the EI concept became famous.

Research studies have clearly stated that emotions induce changes at three levels such as physiological, cognitive, and behavioural, ^2^ as well as that they have both positive and negative reactivity. For example, an individual gets frightened on seeing a snake (negative valence), which will be implied physiologically as an increase in heart beats, cognitively as racing thoughts about danger, and behaviourally as an urge to run away. Researchers and psychologists such as Charles Spearman, Louis L. Thurstone, Joy Paul Guilford, Robert Sternberg, and Howard Gardner defined the nature, structure, characteristics, and types of human intelligence. Some of the famous pioneers in the EI research include John Mayer, Peter Salovey, David Caruso, Daniel Goleman, E.L. Thorndike, and Reuven Bar-On, who have illustrated the significant features that regulate an individual’s EI.

General definition of EI is the ability of an individual to recognize, practice, and regulate emotions in oneself in positive ways to overcome issues, communicate efficiently, understand others, relieve stress and resolve conflicts. Dr. Daniel Goleman, a researcher and psychologist from Harvard University, defined EI as an ability of a person to identify emotions in oneself and others, to motivate oneself, and to handle his or her feelings in a way to express them appropriately and effectively to others. ^3^

EI was defined by Mayer & Salovey (1990) in terms of cognitive abilities/intellectual aspects, where Goleman & Reuven Bar-On described about EI in terms of Personal aspects/Personality traits. The Four-branch model of emotional intelligence proposed by Mayer & Salovey focuses the greatest emphasis on perception, facilitating thoughts with the emotions *i.e.* emotional integration, understanding and managing the emotions. ^4^ On the other hand, the Reuven Bar-On (2002) ^5^ model composed of emotional and social abilities, skills and facilitators, and it relays on the abilities such as awareness of ones’ emotions, understanding interpersonal dynamism, self-actualization, reality testing, happiness, stress endurance, and optimism. All of these elements are believed to be interrelated and work together cooperative. Furthermore, Daniel Goleman (1998) ^6^ lays his emphasis on self-awareness, self-control or self-management, empathizing skills, problem solving, handling conflicts, and leadership skills as the characteristic features of an emotionally intelligent person. Concisely, Reuven Bar-On’s mixed ability model, which emphasises the impact of person’s personality traits on his overall well-being, while Goleman’s model is more focused on professional skills success at workplace. ^7^^,^^8^

However, Daniel Goleman’s book ‘EI – Why it can matter more than IQ’ (1995) ^9^ owes for the current popularity of the EI theory. Following the approval of EI, several constructs have been predicted. The “ability” model of Mayer and Salovey and Goleman and Bar-On’s “mixed” models are the two major approaches that emerged as the result of dynamic research and intense interest in the EI field and called as theoretical models of EI. The primary approach is known as an ability-based model where EI is defined as an ability to process, understand, analyse, assimilate, and control information related to emotion, which are typically evaluated by items or questionnaires which are not used in traditional cognitive tests. The secondary approach is referred as a mixed-model, in which EI is considered as a combination of ability constructs, personality traits, and intrinsic motivation, which are often assessed by self-report or observer-report rating scales. ^10^ This review talks about EI components, significant theories and models of EI, and the most popular EI assessment tools.

## General components of emotional intelligence

In his book, Goleman (1998) ^6^ presents five components of emotional intelligence:

1. **Self-awareness –** This refers to recognizing own emotions and understanding how they affect one’s thoughts and behaviour. By analysing and knowing one’s own strengths and limitations of an individual, it boosts self-confidence. They are usually receptive, accept constructive criticism, and able to learn from difficult situations.
2. **Self-regulation or self-management –** This refers to the ability to control impulsive emotions (feelings or behaviours), exercise restraint and control when expressing their emotions, manage emotions in healthier ways under distress or challenging situations, being proactive, consistency in commitments, and acclimatize to altering circumstances.
3. **Social skills –** This refers to the ability to understand the emotions, needs, and concerns of others, understand their emotional signs, feeling comfortable in social situations, and identify the complex power dynamics in any setting such as group, company, or organization. They have the ability to connect with other people quickly and build trust, and also to gain respect from others.
4. **Empathy –** Empathy is critical in developing and maintaining good relationships as well as managing a successful team or organization. Empathetic people communicate clearly, motivate and influence others, good players in a team, and handle conflict. They respond genuinely to other people’s concerns, and thus help to develop the self-efficacy of people on their team, challenge the self-esteem of those who are unfair, provide constructive criticism, and listen to the demands of needed ones.
5. **Motivation –** This refers to persistent zeal to work consistently toward the goals, and self-motivated individuals are resilient in nature, driven by an inner ambition, and have very high adherence to their commitments and extreme standards in their work quality.

## Major theories on the evolution of EI

### Gardner’s theory of Multiple Intelligence

A robust critic of IQ tests, Gardner (1998) ^11^ questions on the disappearance of IQ tests, whether it is impossible to identify intelligence in an individual or otherwise. These questions awakened the world to a new possibility which has decided that afar from the intellectual prowess, there are other innate or acquired characteristic features in an individual which must be also accounted or considered before assessing their intelligence. Furthermore, Gardner described that a single intelligence test cannot detect the intellectual or non-intellectual capability of an individual, as every person has multiple latent abilities unique in their own way. These abilities cannot be assessed by the conventional methods of testing. Thus, Gardner defined intelligence as “a psychobiological capability to process the incoming information in order to solve issues or to generate ideas or solutions”. He also argued there are multiple intelligences, not one type of general intelligence, and each one of these multiple intelligences are interconnected and functions as an independent system in the brain. In 1983, Howard Gardner’s theory of Multiple Intelligence was revived, ^12^ which was armed with intensive research in psychology, biological sciences, anthropology, and the cultural studies. In 1998, Gardner stated that “human beings possess a sum of relatively independent faculties, which is far important than having a specific amount of intellectual horsepower (or IQ), which can be channelized in one or another direction”. ^12^ He published his findings and beliefs in his book “Frames of mind: The theory of multiple intelligences” which illustrated seven types of intelligences such as linguistic, logical, musical, spatial, kinaesthetic, interpersonal and intrapersonal. The Multiple Intelligence theory makes two major claims:

(i) All these intelligences are found to be universal in human beings.
(ii) All these intelligences are found in varying degrees, and thus exactly the similar combination of these intelligences is not found in two individuals.

### Thorndike theory of social intelligence

When tracing the evolution of the EI theory, the traditional belief that intelligence refers to cognitive abilities such as logical reasoning, abstract thinking, memory and problem solving ^13^ was challenged by E.L. Thorndike in as early as 1920, even before Gardner (1980) or Weschler (1940). Thorndike declared that ‘non-intellective’ elements being equally important and spoke about ‘Social Intelligence’. He authentically discriminated social intelligence from other forms of intelligence. He defined social intelligence as “the ability to understand and manage men and women, boys and girls – to perform wisely in human relations”. ^14^ Essential definition of social intelligence by Thorndike is the ability to recognize emotions, motives, and reason behind behaviours in oneself and others, as well as to empathize with others based on the perceived information. Based on a person’s ability to understand and regulate emotions, Thorndike classified intelligence into three important facets, ^15^ and they are

• Ideas or notions (abstract intelligence),
• Actual items or things (mechanical intelligence), and
• Individuals (social intelligence).

However, the emergence of social intelligence concept promoted the theories that emphasized on identifying other latent skills in a person and certainly switched the way people perceived intelligence. But in itself, social intelligence was not successful or convincing, and also failed to discriminate itself as a discrete form of intelligence. In 1960, Cronbach stated that even after 50 years of research have neither clearly described the social intelligence nor provided the tools to measure these skills. Even, Thorndike himself argued that in the case of presence of single trait that characterize social intelligence, it is still remains to be found. Fortunately, Guilford’s ‘Structure of Intellect model’ in 1967 revived the research in social intelligence, which postulated the significance and importance of social intelligence that was acknowledged by the general public. In 1981, Sternberg and his co-workers listed out the qualities which are considered to be essentially present in an intelligent person. They postulated Sternberg’s triarchic theory of intelligence in which three main parts of intelligence include analytical intelligence ( *e.g.,* problem-solving skills, good decision-making skills), creative intelligence ( *e.g.,* effectively handling new problems using past knowledge, experiences and current skills), and practical intelligence (e.g., adapting to new situations and environments, empathize with others’ needs and concerns, communicating effectively, being socially interested and active). ^15^^–^^17^

### Mayer and Salovey theory of EI

Even though the field of EI is only at its nascent stage, the concept and term ‘EI’ was introduced and coined by Peter Salovey and John Mayer in the initial stages. They used the term ‘EI’ in the literacy writing in in 1990, ^13^ and presented EI as a subset of social intelligence. ^14^ They defined EI as an ability to accurately perceive feelings emotions of one’s own and others’, to differentiate among them, to check, analyse and generate emotions in order to facilitate thought process, to reflectively regulate emotions to guide one’s cognition and action in order to promote emotional and intellectual growth. ^4^ Another definition of EI by Salovey and Mayer is the capacity to imbibe emotions, facilitate emotional feelings with thoughts, processing the emotion-related information, and handle them effectively. ^18^

Apart from discriminating EI from other types of intelligences, they presented a skeletal framework which described a set of skills that helped people to regulate emotions in oneself and others, and also developed ‘ability model’ – the only one to date to analyse EI. Initially, they conceptualized a set of skills such as appraisal and expression of emotion, emotional regulation and utilizing the emotions, which may help in better emotional management. ^19^^,^^20^

(i) **Appraisal and expression of emotion** is defined as an ability of an individual to accurately perceive their emotions as well as decode the expressions of others’ feelings, and also to respond appropriately to others, and thus it is also called as emotional recognition. Appraisal and expression of emotion was further classified as identification of emotion in self ( *e.g.,* usually transmitted through verbal and non-verbal perception) and others ( *e.g.,* usually transmitted through non-verbal perception [ *i.e.,* the face, body, and voice] and empathy). This ability is positively linked to better empathizing skills, adaptability, and social interactive skills. These skills form a critical component of EI, and it demands the accurate processing of emotion-related information in oneself and in others.
(ii) **Emotional regulation** in self and others is defined as an ability to consciously perceive and regulate emotions according to an individual’s will. It allows a person to control their reactions or responses, so that they are not governed by impulsive behaviours and feelings. This ability is positively associated with more flexibility, extraversion, resiliency, and receptivity, and is negatively correlated with criticism on stressful situations and impulsive reactivity to people’s attitudes. This ability also refines a person’s senses in perceiving others’ emotions effectively and thus enables him or her to adapt according to the demands of the situation.
(iii) **Utilizing the emotions** refers to the ability to use emotions for the benefit of oneself and others. When the positive aspects of emotional regulation optimize, it leads to emotional utilization *i.e.,* the constructive use of the emotional arousal energy. This ability is positively correlated with flexible planning, creative thinking, enhanced focus and motivation, and solve problems adaptively. ^14^ These skills put together enables him or her to get the best out of any situation.

## Mayer and Salovey’s ability model

Mayer and Salovey’s four branch model of EI stratified EI into four stages through which a person becomes emotionally intelligent. ^21^

• Emotional perception – This refers to an ability to accurately perceive emotions in oneself and others, and to exhibit them accurately.
• Emotional assimilation - This refers to an ability to differentiate different types of emotions such as basic emotions, discrete emotions, specific emotions, and emotional schemas, and use these emotions to facilitate thinking.
• Emotional understanding - This refers to an ability to recognise emotional nexus and their meanings, and also to identify the conversion from one emotion to another.
• Emotional management - This refers to an ability to handle their emotions by attaching to or detaching from any emotion at a specific situation, which exercise restraint or control over his impulsivity, and thus promoting the ability to think logically, calculate, and act reasonably in any situation. The process of managing emotions is entirely a cognition-based psychological process, and intellectual prowess definitely plays a major role. ^19^^,^^20^

On the other hand, Reuven Bar-On and Goleman projected the mixed ability models which include certain personality traits as well, which include a collection of emotion-related dispositions and self-perceptions. Generally, mixed models of EI are defined as the models that mix together the core idea of EI with a variety of other personality traits which maybe unrelated to emotions or intelligence.

## Daniel Goleman’s theory of EI

In 1983, Howard Gardner explained that his hypothecation of personal intelligences is based on intrapersonal (also known as emotional) intelligence and interpersonal (also known as social) intelligence. Captivated by the research work of Salovey and Mayer, Daniel Goleman (1998) continued his EI research and proposed a four-branch model which in turn included 20 emotional competencies and includes more than 25 characteristics of EI which range from emotional self-awareness to diverse qualities as teamwork, collaborating skills, service orientation, taking initiative, motivating others and formulating goals. Addition of personality traits like trustworthiness, innovation, team player, and other personality features lead to further criticism. ^22^ His contribution in the field of EI is phenomenal, as he popularized the theory to wider audience and emphasized the topic with his book in 1995, ‘EI: Why it can matter more than IQ’. He characterized EI as a latent, inborn latent, and which later lead to the development of emotional competencies. He described emotional competencies as factors which are not innate talents, but those that can be learnt and developed. He compared EI and emotional competencies to apples and apple sauces. ^23^ He pioneered the application of EI concept to business by publishing an article in Harvard Business Review. ^24^ Particularly, individuals high in EI have good interpersonal, social and team building skills, avoid conflicts, fights, and other social altercations, and develop good social interactive skills. For example, If IQ get a placement in an organisation, his EI determines his stability and accomplishments in his workplace. ^25^^–^^27^ Highly intelligent people are more open, receptive, agreeable, extroverts, prevent self-destructive habits or negative behaviours such as smoking, excessive drinking, or drug abuse, and less apt to engage in problem behaviours or violent episodes with others. They tend to be involved in occupations that require social interactions like teaching, coaching, counselling and educating other individuals and they live together with harmony, acceptance, and satisfaction ^28^^,^^29^

## Goleman’s EI competency model

a. Goleman’s 4-branch model included ^28^^,^^29^:
b. Self-awareness cluster include precise self-assessment and self-confidence.
c. Self-management or Self-regulation cluster include self-control, adaptability, trustworthiness, conscientiousness, and innovative skills.
d. Motivation cluster include achievement drive, initiative, commitment, and optimism.
e. Empathy cluster include developing others, understanding others, service minded, balancing diversity social orientation and organizational awareness.
f. Social awareness or social skills cluster include communication, influence, conflict management, team building, teamwork, leadership, change catalyst, bonding, and collaboration.

Goleman established Emotional Competency Inventory (ECI), which is a multi-rater instrument that offers self-report, expert (manager) report, direct report, and associates’ ratings on several behavioural indicators of EI. ^7^ About 40% of the ECI was derived from the previous questionnaire, Self-Assessment Questionnaire, which was developed by Richard Boyatzis (1994) based on the EI competencies. ^30^ EI competencies refer to a measure of competencies which was extracted and verified against the performance of several number of executives, general managers, and leaders in North America. ^31^ ECI was improved upon the Self-Assessment Questionnaire in order to expand the application across all occupations and life settings.

## Bar-On’s theory of EI

Bar-On’ theory incorporates both emotional and social competencies, and referred his model as ‘Emotional Social Intelligence’ (ESI) rather than EI or social intelligence. ^5^ The key concepts related to emotional and social competencies, skills and facilitators referred in Bar-On’s ESI theory include one or more of the following main components: (a) the ability to identify, comprehend and express emotions or feelings; (b) the ability to empathize and relate to other’s feelings and emotions; (c) the ability to regulate and control emotions; (d) the ability to handle alteration, acclimatize and resolve problems of a private and mutual nature; and (e) the ability to create positive affect and remain self-motivated and optimistic. ^32^ He defined ESI as a cross-section of interdependent emotional and social competencies, skills and aids that govern the effectiveness of understanding and expressing emotions in oneself and others, and associate with them, and manage our daily emotional demands. He postulated that EI can be educated and established over a span of time via training, emotional programming in a sequential basis, and therapy. ^7^ He believes that highly intelligent subjects are better in coping skills when they face challenges and pressures of everyday life.

## Bar-On’s (2002) model of EI

Bar-On’s model of EI is considered process-driven rather than outcome-driven approach, as it concentrates mainly on the ‘potential or capacity for success’ rather than ‘success’ itself. ^32^ Further, Bar-On’s model associates EI to positive psychology which correlates positively with a person’s overall happiness and mental well-being. ^5^^,^^33^ Similar to Mayer and Salovey’s model, Bar-On’s model also includes four emotional aspects (self-awareness, self-control, self-expression, and empathy) as well as reality testing—the ability to assess the gap between the actual meaning and his or her emotional experience or perception, and enduring stressful experiences—the ability to remain strong, optimistic and balanced in the face of difficulty. In contrast to Goleman’s model, Bar-On model includes stress handling techniques and mood components like optimism, staying positive, and happiness. Additionally, they join reality testing which also assess the impulse control, an ability in stopping oneself from engaging in a reckless manner to a certain stressful or challenging situation. Bar-On’s (2006) model illustrates the five important components which are further sub-divided into 15 subcomponents ^26^:

• **Intrapersonal components** include sub-components such as self-awareness, self-respect, self-actualization, independence, and assertiveness.
• **Interpersonal components** include sub-components such as prosocial skills, empathy and mutual relationship.
• **Adaptability components** include sub-components such as reality testing, cognitive restructuring, flexibility, and problem-solving.
• **Stress management components** include sub-components such as stress endurance, inhibitory control and impulse control.
• **General mood components** include sub-components such as optimism, humour and happiness.

Based on the theoretic basis of the Bar-On model, EQ Inventory (EQ-i) was developed. EQ-i was formerly devised to assess the various aspects of ESI and also to examine its conceptualization. EQ-i reflected an operationalized form of ESI, which was constructed mainly to examine the functioning of ESI theory. Thus, the EQ-i – a self-report measure which is used to measure the ESI emphases on estimating a person’s coping capacity to the demands and pressures of the situation or environment, ^32^ rather than validating his or her personality or cognitive aspects ^5^ claimed that his model as a ‘better predictor of human performance’ as it enabled to validate both professional and academic aspects ( Table 1).

**Table 1.** The three major conceptual models suggested by encyclopaedia of applied psychology (Spielberger, 2004).

Models name	Definition of the construct	Developed by	References
Salovey-Mayer’s model	This model defined EI as the ability to identify, perceive, handle and utilise emotions which helps to think clearly, and this can be measured by an ability-based measure.	Mayer & Salovey, 1997	^86^
Goleman’s model	This model defined EI as an extensive array of emotional competencies and social skills that helps to achieve higher performance at work, and this can be measured by multi-rater assessment.	Daniel Goleman, 1998	^87^
Bar-On’s model	This model defined EI as a cross-section of correlated emotional and social skills, competencies and aids that has stronger influence on the behaviour, and measured by self-report (1997a, 1997b) within an expansile multimodal approach such as interview and multi-rater assessment.	Reuven Bar-On, 1997b, 2000	^83^

## Methods of classifying EI

The primary method of classifying EI measures was suggested by Petrides and Furnham (2000). They distinguished ability EI and trait EI based solely on whether the test aimed at measuring maximal performance (ability EI) or personality aspects in the form of a self-report questionnaire (trait EI). ^34^^,^^35^ The basic difference in this method of classification relays on the constructs measured as the Ability EI tests measure constructs that are related to a persons’ understanding of emotions and emotional abilities on a theoretical basis, while trait EI questionnaires measure the actual behaviours of a subject in emotionally relevant settings (for example, when a person is exposed to stressful event or challenged by an upset friend) and self-rated capacities. Significantly, the method of measurement defines the key type of the EI tested. For instance, all EI aspects measured by self-report items are termed as **‘trait EI’**, whilst all EI aspects measured by maximal performance items are termed as **‘ability EI’**.

Secondarily, EI measures are classified into three EI ‘streams’. ^36^ In the secondary method of classification, stream 1 involve ability measures and stream 2 includes self-report measures, and both are measured based on Mayer and Salovey’s model. On the other hand, stream 3 include ‘expanded models of EI’, which incorporate certain components that are not found in Salovey and Mayer’s definition (p. 443) and referred to as “mixed” models. Stream 3 encompasses both personality and cognitive behavioural items. Several literature reviews in EI often refers the term “mixed EI” as an EI measure that measure a combination of personality traits, social skills and emotional competencies. ^37^

Three major forms of EI measures such as trait, ability, and mixed, have emerged from a number of conceptual similarities in the majority of measures. Especially, most of the measures can be graded based on their meanings, so that scoring by the researcher or psychologists, can be done in the form of a total “EI score” for the participants as well as scores on multiple facets or subscales. Indeed, the initial EI model proposed by Mayer and Salovey has a robust influence on the expansion of EI measures, as the features in ability, trait and mixed measures of EI are shown to have several conceptual super-impositions.

**Ability EI** – Items/questions used in the assessment of ability EI is similar to those found in the IQ test. ^38^ Similar to Mayer and Salovey’s model, the ability EI measure includes all emotional assessment questionnaire as well as all tests containing ability-type items. On the controversy to trait EI measures, ability measures do not evaluate self-report answers given by the test-takers on various statements, but rather score the answers that can solve emotion-related problems, which can be estimated as correct or incorrect. Generally, ability-based measures generate a good score, which indicates a person’s ability to recognise emotions and its functions. But, since they are tests are generated to test the maximal ability of an individual, and they are not designed to predict typical behaviour and trait-based measures. ^39^ However, ability-based measures are weak, but valid and predict a range of professional attitudes such as professional satisfaction ^40^ and work-related performance. ^37^

**Trait EI** – Overall EI and its subdimensions can be measured by trait-based measures using self-report items. Subjects who scored high in various measures of trait EI were estimated to have very high levels of self-efficacy in terms of emotion-related behaviours and also competency in emotional regulation and management in oneself as well as in others. Further, trait EI nearly measures the characteristic behaviour rather than maximal performance, and they are inclined to provide a good validation of actual behaviours in a variety of emotional settings. ^34^ Recently, meta-analyses reports have shown clear association between trait EI and a range of work-related attitudes such as professional satisfaction and loyalty to their organization or company, ^40^ and work-related performance. ^37^

**Mixed EI** - The term mixed EI originally refers to the combination of ability and trait EI measures, which include items/questionnaires aimed at measuring mixture of traits, social skills and competencies that intersect with other personality measures. Even though these measures consist of self-report items, some of the items use 360-degree forms of assessment which include self-report items combined with multiple associate reports such as supervisors, colleagues and subordinates). ^41^ Some of the measures are especially formulated to substantiate and improve the performance in professional atmosphere and can be used as commercial measure. Emotional competencies are theoretically defined as the capabilities that can be learned and developed in subjects to enhance their social skills and enhance professional success. ^42^ Research studies have confirmed the validity of mixed EI measures as it is a good predictor of multiple emotion-related outcomes such as job satisfaction, dedication to organization, ^40^ and work-related performance. ^37^

## Comparing the different forms of EI measures

Different kinds and numbers of dimensions for the various EI measures were developed by researchers based on the various definitions of the EI construct. ^43^ Mainly, these EI measures use diverse response formats such as self-report, ability, and informant approaches. For instance, ECI and EQ-i, commonly used self-report EI measures, sample a broad range of individual differences, but nearly all of the self-report scales have acceptable dependability rate or loaded with well-established personality dimensions. ^44^ A major limitation of self-report measures is that subjects cannot judge their emotion-related abilities and tendencies truly in a reliable way. ^45^^–^^47^ Furthermore, trait-based measures are susceptible to faking. Participants can easily cheat and gain good scores in EI by selecting answers which are in line with a strategic, socially desirable way.

When discussing the acceptability of the ‘ability model’ over other models of EI, ^4^^,^^48^ suggests that certain mixed models, and the insertion of certain personality and non-intellective elements in the EI construct, create much concern by making exaggerated claims. However, the ability model is postulated as the most promising one among all the EI measures, ^49^ but Mayer and Salovey (2003) also propose that EI tests must be applied with great caution. The ability model recommends a ‘purer’ approach to the theory by making a firm amalgamation of emotions and intelligence cooperatively compared to the mixed ability model. ^50^ Ability-based EI measures ( *e.g.,* Multifactor EI Scale [MEIS] and Mayer-Salovey-Caruso EI Tests Version 2.0 [MSCEIT V.2]) are more discrete from the Big Five personality dimensions, and also have high association with general mental ability than self-report EI measures. ^51^ Additionally, Van Rooy *et al.* (2005) ^48^ analysed the ability model and supported the efficacy of this model over the mixed ability models, as ability model clearly differentiates EI from IQ and other personality models. However, their study indicated a high correlation between self-report and personality measures, while no correlation was found between MSCEIT and either personality or cognitive based measures. These results create serious doubts regarding the validity and reliability of the ability model. The basic advantages of the ability EI measures are that they are not subjected to be faked, as the participants are asked to provide the answers which they feel or believe as correct, and also try to obtain a high score. Furthermore, test-takers are engaged actively in the ability EI measures as they attempt to solve emotion-related problems and puzzles, and rate the emotional states in pictures, in contrast to simply rating the agreements with statements in trait-based measures.

Because of the disadvantages such as overlapping with the cognitive ability and statistical validity in predicting work performance, ability-based EI measures are also considered as the measures of social intelligence. Even Thorndike suggested that early measures of social intelligence tests measured the general mental ability very poorly. ^52^^,^^53^ Among few studies investigating the level of overlap between the trait and ability-based EI measures indicated that the correlation between MSCEIT and Bar-On scales was 0.36, ^54^ and the correlation between the MSCEIT and Bar-On scales was 0.21, ^55^ indicating that they share approximately 13% and 4% of their variance, respectively. The correlation values between different EI measures are evidently low, which gives raise to doubts whether these items are all measuring the same construct. ^56^ Nevertheless, self-report measures often receive lesser attention than ability-based EI measures, because of the lack of psychometric support. On the other hand, ability-based EI measures receive attention continuously, and henceforth, additional assessments conducted to estimate the convergent validity across various EI measures have become essential.

Other major disadvantages of ability-based measures are the questions raised by many personality and intelligence theorists who suspect the existence of ability EI. They postulate that ability EI is similar to intelligence, which is supported by high correlation coefficient score between ability EI and IQ. ^57^ In terms of reliability and validity, ability EI measures commonly have very poor psychometric properties, and do not predict the variables strongly. ^37^^,^^40^ Maul (2012) has reported a comprehensive set of problems with the MSCEIT’s interpretive argument, in terms of consensus-based scoring, lower reliability, and underestimation of the EI construct, and indistinct logical association between the test content and the construct. ^58^

## Emotional intelligence assessment measures

The following EI assessment measures were chosen to be highlighted in this article as they are all utilised regularly, and enable the researchers, psychologists, clinicians, and psychiatrists to measure many of the major features common to EI measures ( *e.g.,* perceiving, regulating, and utilizing emotions).

### Multifactor Emotional Intelligence Scale (MEIS)

In 1997, Salovey and Mayer proposed a four-branch approach to ability EI called Multifactor EI Scale (MEIS), which was developed in an intelligence testing tradition, as they believed EI includes the capacity or ability to rationalize with and about emotions. ^59^ In order to provide a standard criterion to test the EI and accept it as scientifically valid, Mayer *et al*. (1999) ^18^ revised the MEIS and proved that possibility of EI as a unique form of intelligence by providing good existential evidence. The MEIS is an ability test, and includes 402 items, and used a 12-subscale ability test to assess the EI of the participants (particularly in adult and adolescent population). Mainly, 12 different tasks validated the various abilities classified under the four branches:

• Emotional perception – test-takers are asked to detect emotions in faces, music, designs and stories in oneself and others.
• Emotional facilitation of thinking – test-takers are asked to define their emotional feelings and pretend situations where any particular emotion is primarily significant.
• Emotional understanding – test-takers are asked to recognize the blending of two emotions (shock and despair, fear and anxiety *etc.*) and when one emotion changes into another (transition - anger turning into hatred, sadness becoming depression *etc.*).
• Emotional management – test-takers are asked to how they would act when they are given imaginary situations.

The internal consistency reliability of the overall MEIS was 0.95. ^59^ The average internal consistency reliability across the four branch scores was 0.77 for consensus scored scales and 0.62 for expert scored scales. ^56^^,^^60^ The test–retest reliability of the overall MEIS was 0.75 with the interval of 2-weeks, while for the MEIS branch scores, the test–retest reliability ranged from 0.60 to 0.68. The reliability coefficients for cognitive ability tests ranged from 0.85 to 0.95. ^61^ The convergent validity between MEIS and EQ-i was found to be 0.36, displaying that the tests share 13% of their discrepancy. In terms of discriminant validity evidence, the correlation score for the MEIS (consensus scores) with the Big Five personality dimensions was found to be in the range of 0.13 (for Openness and Extraversion) to 0.24 (for Agreeableness). ^50^ For the MEIS branch scores, traditional measures of cognitive ability showed a correlation of 0.30 to 0.40. ^50^^,^^51^ To identify the correct answers, different approaches have been tried and three different scoring methods such as target, consensus, and expert scoring methods are applied. The participant’s task is to answer questions about cognitions, emotions, personality, and relationships either by putting themselves in the place of the target person or by making a self-evaluation of the target. Target scoring involves asking the person about the correct emotional expression or feeling depicted in an item by the target when he or she was exposed to specific emotional situations. Consensus scoring involves defining the correct answer by including the answers of test takers. ^56^ Expert scoring involves deciding the correct answer by including the answers of 15 experts representing three different occupational groups. However, the limitations of MEIS scale were low reliability for some subscales and few problems with scoring procedures.

### Mayer-Salovey-Caruso Emotional Intelligence Tests (MSCEIT)

The MEIS-related problems were successfully addressed and resolved in Mayer’s 2003 revision of the MSCEIT, which also improvised the items/Questionnaire to determine whether there are correct answers to the tasks given. ^59^ According to the statement of Legree (2005) ^61^ asserts that ‘accumulation of experts (above two) is necessary to achieve a reliable identification of answers’ and thus as a result MEIS was enhanced by bringing in 21 emotions experts. The MSCEIT was found to be highly reliable, and its factorial structure and correlation demonstrated positive intercorrelation between all the tasks. Following, several additions were revised later and MSCEIT V.2 was developed ^60^ (p0).

The MSCEIT V.2 is an updated version of MEIS that also measures the four branches of Mayer and Salovey’s (1993, 1997) EI ability model. ^9^^,^^16^ This updated model is a process-oriented model that emphasises the stages of EI developmental, and how EI correlates with the development of potential for growth, and how emotional understanding facilitate the intellectual development. Each of the four branches is measured with two objectives, ability-based tasks Branch. ^62^ Response formats varied because some tasks utilised multiple-choice response such as the “blends tasks” while others used 5-point rating scales such as the “picture task”, similar to scoring in the IQ tests, where correct or incorrect answers can be measured and tailed for all questions. The MSCEIT V.2 describes a total EI score as four Branch scores: 1. Emotional perception – representing the ability to identify the exact feelings in oneself and others, 2. integration and assimilation of emotion - represents the ability to modulate/regulate emotions that affects the thought process, 3. knowledge about emotions - indicates the ability to understand the reasons for emotional arousals, and 4. management of emotions - represents the ability to formulate effective strategies or techniques that utilise emotions for a particular purpose. Since MSCEIT V.2 is a condensed version of MEIS, it can be administered more quickly than MEIS, and it provides consensus and expert scores for all Branch scores and has of eight tasks with total of 141 individual items in total and four constructs. Where the Branch levels and total scale reliabilities were more than 0.75. ^63^ For consensus and expert scoring for all scales, the average internal consistency reliability were 0.68 and 0.71, respectively. However, the subscale reliabilities were extremely far from their ideal values. ^56^ To support the MSCEIT V.2, validity measures were relayed primarily on evidential data from the MEIS, caution is required when deriving conclusions about MSCEIT V.2.

## Self-report Emotional Intelligence Test (SREIT)

In 1998, Schutte developed a self-report EI questionnaire, called as Schutte Self-Report EI Test (SREIT) based on Salovey and Mayer’s model (1990). ^64^ The SREIT is a self-report test of emotional intelligence that tested four EI constructs such as appraisal and expression of emotion, optimism or management of emotion, social skills and utilisation of emotions. ^64^ SREIT consists of 33 self-report questionnaire items which were designed in order to assess the four scales among the several EI constructs such as “emotional appraisal (perceiving emotions in self), social skills (regulating emotions in others), optimism or mood regulation (regulating emotions in self when facing challenges or adversity), and uses of emotions (strategically utilizing emotions to resolve issues and improve mood and well-being)”. The test’s internal consistency was strong (exhibiting Cronbach’s alpha of 0.90) and correlation for test-retest reliability was also found to be 0.78. The scale was also designed to test against theoretically related constructs such as “alexithymia, optimism, pessimism, mood regulation, non-verbal communication of affect, recognition and clarity of feelings, depressive symptoms and impulsive behaviour”. Petrides and Furnham (2000) criticised the SREIT scale, even though it has good internal consistency and test-retest reliability, it was criticised for its one-dimensional structure such as trait forms of EI and the psychometric properties such as confusing ability. ^34^ Additional criticism was acknowledged by the other authors, and then was confirmed by O’Connor and Athota, 2013. ^65^ Subjects were instructed to answer the questionnaire items on a 5-point Likert-type scale ranging from 1 point (denotes strong disagreement) to 5 point (denotes strong agreement).

## Trait Emotional Intelligence Questionnaire (TEIQue)

The TEIQue is accessible in long form (153 items, 15 facets, and 4 factors) and short form (30 items, and 4 factors or subscales). ^66^^–^^68^

**TEIQue-Long forms** – As EI is largely determined by human personality, inclusion of personality aspects provide the context in which EI operates. Comprehensively, the TEIQue is based on trait EI theory, and designed as self-report inventory. This theory conceptualizes EI as personality trait, which also described it as “emotional self-efficacy”. In contrast to Schutte *et al.* (1998) ^64^ measure, it is not designed to validate ability-based EI using the self-report questionnaire. It comprises 153 items, estimating 15 distinct facets, 4 factors, and global trait EI. ^69^ A thorough analysis of the EI literature and available constructs ^14^^,^^41^^,^^70^ together led to the development of these items and facets.

**TEIQue-Short forms –** Petrides and Furnham also developed a short-form questionnaire. This short-form questionnaire contains 30-items and the same four factors (such as self-control, well-being, sociability, and emotionality) similar to the long version, which is designed mainly to estimate the global trait EI (trait EI). From each of the 15 facets of the TEIQue, only two items were chosen based on the correlations with corresponding total facet scores for the inclusion in the short form. ^71^^,^^72^ In the short form scale, it is often recommended that all factors or subscales are used as they detect results in different ways. ^39^ All subscales carry equal importance and should be included. ^73^^,^^74^

**TEIQue 360° and 360°-Short Form –** These are the other adaptable forms of TEIQue which are utilised for the assortment of other-ratings. Both the long- and the short-forms of the TEIQue have their own 360° form. Particularly, they are useful when the results for self-versus observer-ratings contrast on trait EI (examples like in leadership skill research and its applications). ^71^

## Bar-On Emotional Quotient Inventory (EQ-i)

The EQ-i model was developed by Bar-On ^41^ by mixing cognitive ability with personality aspects, as he considered EI as a mixture of intelligence and personality *i.e.*, mixed construct. The EQ-i scales put emphasis on the influence of personality traits on a subject’s overall well-being. Bar-On’s model was developed from data obtained from empirical research which indicate that personal factors related to EI influence the emotional and social elements of behaviour particularly. The EQ-I is intended to measure the abilities and the potentiality to perform rather than performance itself, and thus EQ-i can be considered as process-oriented measure rather than outcome-oriented measure. The EQ-i consists of 133-self-report assessment items, which takes around 25-30 minutes to complete. ^75^ The EQ-i scoring is provided as a total score as well as factor and facet/subscale scores. The scores for five composite scales such as intrapersonal, interpersonal, adaptability, general mood status, and stress management were measured individually. Revision on the EQ-i scale led to the development of EQ-i 2.0. ^75^ The overall EQ-i can be used to provide total EI scores and also subscale scores.

The overall internal consistency reliability of EQ-i was 0.76. The test–retest reliability of the EQ-I was adequate, which was found to be 0.85 after 1 month and 0.75 after 4 months. ^41^ Comparable to correlations in traditional intelligence tests and its various components, EQ-i showed the average correlation between various EQ-i subscales was found be aggregable ( *i.e.*, 0.50 with respect to convergent validity). ^43^ The correlation of the EQ-i was 0.36 with the MEIS. ^54^ In terms of the discriminant validity, ^76^ the correlation score of the EQ-I with the Wechsler Adult Intelligence Scale was found to be 0.12, ^75^ while the average EQ-i correlation score with the Big Five personality measures was found to be around 0.50. The correlation between the EQ-i and the anxiety scale from Cattell’s 16PF test was found to be -0.77, indicating strong overlap of EI measure with a trait anxiety measure. ^77^ In terms of criterion validity (Slaski and Cartwright, 2002), a study conducted on retail managers showed an EQ-i correlation of 0.55, -0.41, -0.50, and 0.22 with morality, stress, general health, and supervisor performance ratings, respectively. Indeed, Bar-On (1997) also proposed that the EQ-i measures non-cognitive features of individuals’ performance. For example, a student’s ability to handle everyday pressures and demands. Conclusively, Bar-On (1997) postulated that EI is an important determinant of academic success, based on the items included in non-cognitive aspects, which was supported by the unpublished studies mentioned in the EQ-i Technical Manual. The main limitations of EQ-i are the lack of discriminant validity evidence despite of its adequate reliability and some validity evidence. The correlation coefficient of the total EQ-i score was only 0.01 with their Grade Point Average in a study conducted on 160 Canadian college students, ^77^ which unqualifies the EQ-i as a selection device. Similar to Goleman’s ESCI model, Bar-On’s model is subjected to be faked, as the participants can intentionally tailor their answers for better scores. ^78^ Therefore, further validation for the constructs is necessary.

## The Situational Test of Emotional Management (STEM) and The Situational Test of Emotional Understanding (STEU)

MacCann and Roberts (2008) ^79^ developed ability-based EI measures. Mainly in this model, the STEM and the STEU scales assess the strategic EI areas such as understanding and managing emotions ^19^^,^^20^ out of the four hierarchical ordered branches of emotion-correlated abilities outlined by Mayer *et al.* previously (2000a, b).

**STEM:** The STEM consists of 44 multiple-choice items, and it was designed in both multiple-choice and rate-the-extent formats (such as the participants need to appropriately rate the relevancy, strength, or extent of each alternative, rather than choosing the correct answer). A pilot study investigating the emotional states of 50 individuals, which they had described from their past two weeks experiences (with a total of 290 situations) using a semi-structured interview method were used to assign the questionnaires or items in the STEM model. These items were designed by conducting semi-structured interviews with. These items were categorized such as 18 items for anger, 14 items for sadness, and 12 items for fear and tested in individuals. Estimates of Cronbach’s alpha were slightly low (0.68) in a study conducted using 207 Australian students ^80^ and reasonable (0.85) in a study conducted using 850 Belgian medical students. ^79^ Some evidence of convergent and discriminative validity of the STEM was found. The correlation validity between the STEM and MSCEIT management scores was found to be 0.30, and were also found to correlate with understanding and perception of emotions. ^38^^,^^79^^,^^80^

**STEU:** The STEU is an ability test for emotional understanding, a key component of EI. STEU assesses the understanding the emotions of oneself and others, and the reason behind the evolution of different emotions in complex situations. This test is developed based on ability models and more suitable than self-report model in order to prevent overlapping with personality traits. The items in the STEU model were constructed based on the Roseman’s (2001) emotion appraisal theory, and thus the scoring is done by marking and adding the number of the correct or incorrect answers. In this model, combination of seven appraisal dimensions explain the 17 most common emotions. In the standard version, the STEU scale consists of 42 multiple-choice item stems with five possible reactions. The STEU has 19 scenarios selected from 42-item version of STEU using an inter-response time analysis. ^79^^,^^81^ In each questionnaire item, a different interpersonal scenario presenting emotional situations is described, the participants are asked to select the more likely emotional response experienced by one of the persons in that situation from five of the given emotions. In this model, 14 emotions were evaluated in three separate contexts – de-contextualization or context-reduction (n =14 items), work or workplace context (n =14 items) and private or personal life context (n =14 items).

## Emotional and Social competence Inventory (ESCI)

Boyatzis, Goleman and his co-workers developed an ESCI model which is a mixed model of EI. ^31^ They designed ESCI to evaluate both the personality aspects and cognitive abilities in the perspective of emotional competencies and positive social behaviours. ^31^^,^^70^ This model emphases mainly on predicting workplace success. The ECI has 110 items and evaluates 20 competencies, which are classified into four clusters: (a) Self-Awareness, (b) Self-Management, (c) Social Awareness, and (d) Social Skills. The ESCI also utilises 360-degree assessment techniques such as self, peer, and supervisor ratings. Boyatzis and Goleman embed a set of emotional competencies within each construct of EI In ESCI model. They defined emotional competencies are not inbuilt capabilities, but as abilities that can be learned and applied in activities by an individual to attain an outstanding performance. However, they also argued that generally individuals need to have an innate EI potential that determine their ability for learning emotional competencies. The internal consistency reliability of the self-assessment ESCI scales was found to be in the range of 0.61 to 0.85, while for peer and supervisor rating scales was found to be in the range of 0.80 to 0.95. ^43^ The validity evidence of ESCI is derived from the Self-Assessment Questionnaire, which is a precursor of the ESCI. Few independent, peer-reviewed research studies that assessed the reliability (accuracy) and validity of the ESCI have produced uncertain findings. Limitation of ESCI model include content overlap of the ESCI competencies with the four dimensions of the Big Five personality Inventory such as openness to new experiences, extraversion, conscientiousness, and emotional stability, and other psychological constructs such as motivation and leadership qualities. ^51^^,^^56^

## Discussion

The EI concept has gained much attention from 1990 from the psychological society, academic areas, therapy settings and mainstream society. EI consists of several constructs which are devised to measure an individual’s different traits or abilities. Overall, research studies conducted for more than a decade based on EI-based theories and models have demonstrated the importance and crucial role of EI in the overall intelligence, and its positive correlation with good mental health and well-being. ^82^

Several theories have been proposed by various researchers and psychologist from 1990 till date. Among them, the most rational and famous theories and models are described in this review. First, Howard Gardner postulated the multidimensional aspect of intelligence, which cannot be measured by single intelligence test. ^12^ Second, non-intellectual skills are also equally important for the success and well-being of individual’s life in addition to intellectual skills, is the central point of E.L. Thorndike, who proposed Thorndike theory of social intelligence. ^15^ Third, Peter Salovey and John Mayer proposed EI as a subset of social intelligence. They described the concept in detail and coined the term, and proposed ability model. ^14^ Forth, Daniel Goleman (1998) gave a new perspective to EI by deeply explaining the nature, evolution, biology and importance of EI. His proposal popularized the EI concept worldwide, ^24^ as he included several characteristics of EI from emotional self-awareness to social skills, job performance, teamplay, taking initiative and motivation. Finally, the two broad models of adult EI proposed by Bar-On ^32^^,^^83^ and Mayer and Salovey ^19^^,^^21^ correlate with different skills of emotional competence. Specifically, emotional self-awareness, emotional perception in oneself and others, ability to empathize with others’ feelings and concerns, management of emotional response and modulation of emotional reactivity are found to be related both to EI and emotional competence skills.

Although, the type of EI measured depends on the method of measurement used and is intricated by the different results obtained through the ability and trial EI, without any correlation. Nevertheless, ability, trait and mixed EI measures help in understanding, analysing and handling emotions in oneself and others, which in turn associated with an improved quality of life. Even though the theory and models predict the EI score, in terms of operationalisation, developers and theorists posit the need to design an instrument or a tool (consisting a list of questionnaire or items related to emotional, personality and social competencies) or improve the existing measures to precisely evaluate and assess the emotional skills of a subject. ^84^ The pure models (ability or trait) highlight the cognitive ability, and measures the scores using an objective, performance-based test, while mixed models evaluate IQ and personality traits using self-report questionnaire.

Measures of ability EI are found to overlap partially with long-standing measures of coping approaches and emotional regulation; but some empirical research postulates that ability EI predicts additional information that can be learned through personality and coping measures. ^49^^,^^50^ On the contrary, trait EI measures are found to overlap significantly with long-standing, well-defined measures of personality, and so its use and added applicability value is currently under debate. However, a few models are found to provide a good indication of EI and social skills. For example, Bar-On model conceptualized ESI as a multifaceted collection of interconnected emotional and social competencies, skills and facilitators. These competencies are found to impact one’s ability (a) to identify, comprehend and manage emotions, (b) to empathize with others, (c) to acclimatize to variation and resolve problems, and (d) to deal with everyday demands, tasks and burdens. Although the significance and utility of ESI has been revealed by investigating its ability to indicate different features of behaviour, its predictability for emotionally and socially intelligent behaviours are limited, as there are only certain number of questions which evaluate the emotional states and social skills of a test-taker in a specific situation. By developing an expanded model of ESI, these limitations can be handled, and more comprehensive and exclusive measurements can be derived by constructing a multi-tiered and multi-channel approach. The best-known measures such as the EQ-i, the ESCI, and the MSCEIT are found to be quite expensive, and thus their use regularly in a university or clinical setting is quite difficult. Many other EI measures are available freely with adequate reliability and validity similar to the better-known measures, ^85^ which can used extensively in any setting.

In spite of the extensive research and data collected on EI in the last decade, EI still remains a contentious topic. The number of the constructs involved in EI, the validity of the construct, advantage of one type of model over another, the type of EI measures, and the ability to teach EI are the frequently debated topics. Overall, three important areas require more focus in EI research. First, the reliability of the each of the models of EI, clear distinction between EI constructs and its related concepts such as personality, and further validation of the developed model needs to be evaluated. Particularly, the ability to learn and develop EI skills is an important arena for future research, and thus the programs designed to teach EI in organizations needs to be evaluated carefully. Finally, further research needs to focus on the implementation of EI construct to public sectors. Henceforth, EI concepts can be applied pragmatically with children and adolescent populations if it can be proven that these concepts can be developed as well as learning these concepts promotes favourable outcomes ( *e.g.,* academic, personal, moral/ethical). ^7^ Over the past two decades, though much is decoded in EI, but still significant questions regarding constructs, measuring tools, and the capacity to learn and apply EI in reaching desirable outcomes remains to be uncovered. ^10^

## Data Availability

No data are associated with this article.

## References

[1] Woodworth RS : *Psychology: Fourth Edition.* 4th Edition/Unstated Printing. Henry Holt & Company;1940 [cited 2022 Jul 19]. Reference Source

[2] Lewis M : Self-conscious emotions: Embarrassment, pride, shame, and guilt. *Handbook of emotions.* 3rd ed. New York, NY, US: The Guilford Press;2008; pp.742–756.

[3] Cherniss C Extein M Goleman D : Emotional Intelligence: What Does the Research Really Indicate? *Educ. Psychol.* 2006 Dec 1;41(4):239–245. 10.1207/s15326985ep4104_4

[4] Mayer JD Salovey P Caruso DR : Emotional intelligence: Theory, findings, and implications. *Psychol. Inq.* 2004;15(3):197–215. 10.1207/s15327965pli1503_02

[5] Bar-On R : The Bar-On Model of Emotional-Social Intelligence. *Psicothema.* 2006 Feb 1;18 Suppl:13–25. 17295953

[6] Goleman D : The emotional intelligence of leaders. *Lead. Lead.* 1998;1998(10):20–26. 10.1002/ltl.40619981008

[7] Stys Y Brown SL : A Review of the Emotional Intelligence Literature and Implications for Corrections. undefined. 2004 [cited 2022 Jul 19]. Reference Source

[8] Caruso DR Mayer JD Salovey P : Relation of an ability measure of emotional intelligence to personality. *J. Pers. Assess.* 2002 Oct;79(2):306–320. 10.1207/S15327752JPA7902_12 12425393

[9] Goleman D : *Emotional intelligence.* New York, NY, England: Bantam Books, Inc;1995; vol.xiv:352. (Emotional intelligence).

[10] Mayer JD Salovey P Caruso DR : Emotional intelligence: new ability or eclectic traits? *Am. Psychol.* 2008 Sep;63(6):503–517. 10.1037/0003-066X.63.6.503 18793038

[11] Gardner H : A multiplicity of intelligences. *Sci. Am.* 1998;9(4):19–23.

[12] Gardner HE : *Frames of mind: The theory of multiple intelligences.* Basic books;2011.

[13] A Multiplicity of Intelligences: In tribute to Professor Luigi Vignolo - PDF Free Download. [cited 2022 Jul 20]. Reference Source

[14] Cherniss C : Emotional intelligence: What it is and why it matters. 2000 Jan 1.

[15] Salovey P Mayer JD : Emotional Intelligence. *Imagin. Cogn. Pers.* 1990 Mar 1;9(3):185–211. 10.2190/DUGG-P24E-52WK-6CDG

[16] Kihlstrom JF Cantor N : Social Intelligence. Sternberg RJ Kaufman SB , editors. *The Cambridge Handbook of Intelligence.* Cambridge: Cambridge University Press;2011 [cited 2022 Jul 20]; pp.564–581. (Cambridge Handbooks in Psychology). Reference Source

[17] Sternberg RJ : *Beyond IQ: A Triarchic Theory of Human Intelligence.* CUP Archive;1985;436.

[18] Sternberg RJ : The Theory of Successful Intelligence. *Rev. Gen. Psychol.* 1999 Dec 1;3(4):292–316. 10.1037/1089-2680.3.4.292

[19] Mayer JD Caruso DR Salovey P : Emotional intelligence meets traditional standards for an intelligence. *Intelligence.* 1999 Dec 1;27(4):267–298. 10.1016/S0160-2896(99)00016-1

[20] Mayer JD Salovey P Caruso DR : Emotional intelligence as a standard intelligence. *Emot Wash DC.* 2001 Sep;1(3):232–242. 10.1037/1528-3542.1.3.232 12934682

[21] Mayer JD : A field guide to emotional intelligence. *Emotional intelligence in everyday life: A scientific inquiry.* New York, NY, US: Psychology Press;2001; pp.3–24.

[22] Mayer J Salovey P : What is emotional intelligence? *Emot. Dev. Emot. Intell. Implic. Educ.* 1997 Jan 1. Reference Source

[23] Locke E : Why Emotional Intelligence is an Invalid Concept. *J. Organ. Behav.* 2005 Jun 1;26:425–431. 10.1002/job.318

[24] Apples and Applesauce - Emotional Intelligence Consortium:[cited 2022 Jul 20]. Reference Source

[25] Goleman D : What Makes a Leader? *Harv. Bus. Rev.* 2004 Jan 1 [cited 2022 Jul 20]. Reference Source

[26] Emotional Intelligence - Issues and Common Misunderstandings:[cited 2022 Jul 20]. Reference Source

[27] Developing Emotionally Intelligent Organizations: Richard E. Boyatzis and Ellen Van Oosten. July 10, PDF Free Download. [cited 2022 Jul 20]. Reference Source

[28] Bringing Emotional Intelligence to the Workplace: A Technical Report Issued by the Consortium for Research on Emotional Intelligence in Organizations:[cited 2022 Jul 20]. Reference Source

[29] An EI-Based Theory of Performance:[cited 2022 Jul 20]. Reference Source

[30] Boyatzis R McKee A : Intentional change. *J. Organ. Excell.* 2006;25(3):49–60. 10.1002/joe.20100

[31] Goleman D : Emotional Intelligence: Issues in Paradigm Building From the book The Emotionally Intelligent Workplace. 2002 [cited 2022 Jul 20]. Reference Source

[32] Boyatzis RE Goleman D Rhee KS : Clustering competence in emotional intelligence: Insights from the Emotional Competence Inventory. *The handbook of emotional intelligence: Theory, development, assessment, and application at home, school, and in the workplace.* San Francisco, CA, US: Jossey-Bass;2000; pp.343–362.

[33] Bar-On R : The Bar-On Emotional Quotient Inventory (EQ-i): Rationale, description and summary of psychometric properties. *Measuring emotional intelligence: Common ground and controversy.* Hauppauge, NY, US: Nova Science Publishers;2004; pp.115–145.

[34] Bar-On R : Emotional Intelligence: An Integral Part of Positive Psychology. *South Afr. J. Psychol.* 2010 Mar 1;40:54–62. 10.1177/008124631004000106

[35] Petrides KV Furnham A : On the dimensional structure of emotional intelligence. *Personal. Individ. Differ.* 2000 Aug 1;29(2):313–320. 10.1016/S0191-8869(99)00195-6

[36] Pérez JC Petrides KV Furnham A : Measuring Trait Emotional Intelligence. *Emotional intelligence: An international handbook.* Ashland, OH, US: Hogrefe & Huber Publishers;2005; pp.181–201.

[37] Ashkanasy NM Daus CS : Rumors of the death of emotional intelligence in organizational behavior are vastly exaggerated. *J. Organ. Behav.* 2005;26(4):441–452. 10.1002/job.320

[38] O’Boyle EH Jr Humphrey RH Pollack JM : The relation between emotional intelligence and job performance: A meta-analysis. *J. Organ. Behav.* 2011;32(5):788–818. 10.1002/job.714

[39] Austin EJ : Measurement of ability emotional intelligence: results for two new tests. *Br. J. Psychol. Lond. Engl. 1953.* 2010 Aug;101(Pt 3):563–578. 10.1348/000712609X474370 19843352

[40] O’Connor P Nguyen J Anglim J : Effectively Coping With Task Stress: A Study of the Validity of the Trait Emotional Intelligence Questionnaire-Short Form (TEIQue-SF). *J. Pers. Assess.* 2017 Jun;99(3):304–314. 10.1080/00223891.2016.1226175 27690638

[41] Miao C Humphrey RH Qian S : A meta-analysis of emotional intelligence and work attitudes. *J. Occup. Organ. Psychol.* 2017;90(2):177–202. 10.1111/joop.12167

[42] Emotional Quotient-Inventory - PsycNET:[cited 2022 Jul 22]. Reference Source

[43] Goleman D : *Emotional intelligence.* Bantam 10th anniversary hardcover. New York: Bantam Books;2006;358.

[44] Wiley.com: *The Emotionally Intelligent Workplace: How to Select For, Measure, and Improve Emotional Intelligence in Individuals.* Groups, and Organizations|Wiley;[cited 2022 Jul 28]. Reference Source

[45] Daus C Ashkanasy N : Will the Real Emotional Intelligence Please Stand Up? On Deconstructing the Emotional Intelligence “Debate”. *Ind.-Organ. Psychol.* 2003 Jan 1;41.

[46] Brackett M Rivers S Shiffman S : Relating emotional abilities to social functioning: a comparison of self-report and performance measures of Emotional Intelligence. *J. Pers. Soc. Psychol.* 2006 Nov 1;91:780–795. 10.1037/0022-3514.91.4.780 17014299

[47] Sheldon OJ Dunning D Ames DR : Emotionally unskilled, unaware, and uninterested in learning more: Reactions to feedback about deficits in emotional intelligence. *J. Appl. Psychol.* 2014;99(1):125–137. 10.1037/a0034138 23957689

[48] Boyatzis RE : The Behavioral Level of Emotional Intelligence and Its Measurement. *Front. Psychol.* 2018 Aug 13;9:1438. 10.3389/fpsyg.2018.01438 30150958 PMC6099111

[49] Mayer J Roberts R Barsade S : Human Abilities: Emotional Intelligence. *Annu. Rev. Psychol.* 2008 Feb 1;59:507–536. 10.1146/annurev.psych.59.103006.093646 17937602

[50] Conte JM : A review and critique of emotional intelligence measures. *J. Organ. Behav.* 2005;26(4):433–440. 10.1002/job.319

[51] Roberts RD Zeidner M Matthews G : Does emotional intelligence meet traditional standards for an intelligence? Some new data and conclusions. *Emot. Wash. DC.* 2001 Sep;1(3):196–231. 10.1037/1528-3542.1.3.196 12934681

[52] Van Rooy DL Viswesvaran C : Emotional intelligence: A meta-analytic investigation of predictive validity and nomological net. *J. Vocat. Behav.* 2004 Aug 1;65(1):71–95. 10.1016/S0001-8791(03)00076-9

[53] Thorndike RL : Factor analysis of social and abstract intelligence. *J. Educ. Psychol.* 1936;27(3):231–233. 10.1037/h0059840

[54] Thorndike RL Stein S : An evaluation of the attempts to measure social intelligence. *Psychol. Bull.* 1937;34(5):275–285. 10.1037/h0053850

[55] Mayer JD Salovey P Caruso D : Models of emotional intelligence. *Handbook of intelligence.* New York, NY, US: Cambridge University Press;2000; p.396–420.

[56] Brackett M Mayer J : Convergent, Discriminant, and Incremental Validity of Competing Measures of Emotional Intelligence. *Personal. Soc. Psychol. Bull.* 2003 Oct 1;29:1147–1158. 10.1177/0146167203254596 15189610

[57] Matthews G Zeidner M Roberts RD : *Emotional Intelligence: Science and Myth.* MIT Press;2004;724.

[58] MacCann C Joseph DL Newman DA : Emotional intelligence is a second-stratum factor of intelligence: evidence from hierarchical and bifactor models. *Emot Wash DC.* 2014 Apr;14(2):358–374. 10.1037/a0034755 24341786

[59] Maul A : The Validity of the Mayer–Salovey–Caruso Emotional Intelligence Test (MSCEIT) as a Measure of Emotional Intelligence. *Emot. Rev.* 2012 Oct 1;4(4):394–402. 10.1177/1754073912445811

[60] Mayer J Cobb C : Educational Policy on Emotional Intelligence: Does It Make Sense? *Educ. Psychol. Rev.* 2000 Jun 1;12:163–183. 10.1023/A:1009093231445

[61] Kaplan RM Saccuzzo DP : *Psychological Testing: Principles, Applications, and Issues.* Cengage Learning;2012;755.

[62] Mayer JD Salovey P Caruso DR : Measuring emotional intelligence with the MSCEIT V2.0. *Emot Wash DC.* 2003 Mar;3(1):97–105. 10.1037/1528-3542.3.1.97 12899321

[63] Legree PJ Psotka J Tremble T : Using Consensus Based Measurement to Assess Emotional Intelligence. *Emotional intelligence: An international handbook.* Ashland, OH, US: Hogrefe & Huber Publishers;2005; pp.155–179.

[64] Salovey P Mayer J Caruso D : Measuring emotional intelligence as a set of abilities with the Mayer-Salovey-Caruso Emotional Intelligence Test. *Handb. Posit. Psychol. Assess.* 2003 Jan 1. J Lopez C R Snyder Eds. 10.1037/10612-016

[65] Schutte NS Malouff JM Hall LE : Development and validation of a measure of emotional intelligence. *Personal. Individ. Differ.* 1998 Aug 1;25(2):167–177. 10.1016/S0191-8869(98)00001-4

[66] O’Connor PJ Athota VS : The intervening role of Agreeableness in the relationship between Trait Emotional Intelligence and Machiavellianism: Reassessing the potential dark side of EI. *Personal. Individ. Differ.* 2013 Oct 1;55(7):750–754. 10.1016/j.paid.2013.06.006

[67] Petrides KV Furnham A : Trait emotional intelligence: psychometric investigation with reference to established trait taxonomies. *Eur. J. Personal.* 2001 Nov 1;15(6):425–448. 10.1002/per.416

[68] Freudenthaler H Neubauer A Gabler P : Testing the Trait Emotional Intelligence Questionnaire (TEIQue) in a German - speaking sample. *Personal. Individ. Differ.* 2008 Nov 1;45:673–678. 10.1016/j.paid.2008.07.014

[69] Mikolajczak M Luminet O Leroy C : Psychometric properties of the Trait Emotional Intelligence Questionnaire: factor structure, reliability, construct, and incremental validity in a French-speaking population. *J. Pers. Assess.* 2007 Jun;88(3):338–353. 10.1080/00223890701333431 17518555

[70] Petrides K : *Trait Emotional Intelligence Questionnaire (TEIQue): Technical Manual.* London Psychometric Laboratory;2009;91.

[71] Goleman D : *Emotional Intelligence: Why it Can Matter More Than IQ: & Working with Emotional Intelligence.* Bloomsbury;2004;735.

[72] Petrides K Furnham A : The Role of Trait Emotional Intelligence in a Gender-Specific Model of Organizational Variables1. *J. Appl. Soc. Psychol.* 2006 Feb 1;36:552–569. 10.1111/j.0021-9029.2006.00019.x

[73] Cooper A Petrides KV : A psychometric analysis of the Trait Emotional Intelligence Questionnaire-Short Form (TEIQue-SF) using item response theory. *J. Pers. Assess.* 2010 Sep;92(5):449–457. 10.1080/00223891.2010.497426 20706931

[74] Mavroveli S Petrides K Shove C : Validation of the construct of trait emotional intelligence in children. *Eur. Child Adolesc. Psychiatry.* 2008 Jul 1;17:516–526. 10.1007/s00787-008-0696-6 18563477

[75] Mavroveli S Petrides K Sangareau Y : Relating trait emotional intelligence to objective socioemotional outcomes in childhood. *Br. J. Educ. Psychol.* 2008 Nov 1;79:259–272. 10.1348/000709908X368848 18950549

[76] Bar-On R Brown J Kirkcaldy B : Emotional expression and implications for occupational stress: An application of the Emotional Quotient Inventory (EQ-i). *Personal. Individ. Differ.* 2000 Jun 1;28:1107–1118. 10.1016/S0191-8869(99)00160-9

[77] Dawda D Hart SD : Assessing emotional intelligence: reliability and validity of the Bar-On Emotional Quotient Inventory (EQ-i) in university students. *Personal. Individ. Differ.* 2000 Apr 1;28(4):797–812. 10.1016/S0191-8869(99)00139-7

[78] Newsome S Day A Catano V : Assessing the Predictive Validity of Emotional Intelligence. *Personal. Individ. Differ.* 2000 Dec 1;29:1005–1016. 10.1016/S0191-8869(99)00250-0

[79] Grubb WL III McDaniel MA : The Fakability of Bar-On’s Emotional Quotient Inventory Short Form: Catch Me if You Can. *Hum. Perform.* 2007;20(1):43–59. 10.1080/08959280709336928

[80] MacCann C Roberts RD : New paradigms for assessing emotional intelligence: theory and data. *Emot. Wash. DC.* 2008 Aug;8(4):540–551. 10.1037/a0012746 18729584

[81] Libbrecht N Lievens F : Validity evidence for the situational judgment test paradigm in emotional intelligence measurement. *Int. J. Psychol. J. Int. Psychol.* 2012;47(6):438–447. 10.1080/00207594.2012.682063 22823103

[82] Allen VD Weissman A Hellwig S : Development of the situational test of emotional understanding – brief (STEU-B) using item response theory. *Personal. Individ. Differ.* 2014 Jul 1;65:3–7. 10.1016/j.paid.2014.01.051

[83] Ciarrochi J Forgas JP Mayer JD : *Emotional intelligence in everyday life: a scientific inquiry.* Philadelphia, PA: Psychology Press;2001;230.

[84] Monica 1776 Main Street Santa, California 90401-3208: Bar-On Emotional Quotient Inventory: Youth Version (Short) (Bar-OnEQ-i:YV(S)). [cited 2022 Jul 29]. Reference Source

[85] O’Connor PJ Hill A Kaya M : The Measurement of Emotional Intelligence: A Critical Review of the Literature and Recommendations for Researchers and Practitioners. *Front. Psychol.* 2019 May 28;10:1116. 10.3389/fpsyg.2019.01116 31191383 PMC6546921

[86] Jensen S Kohn C Rilea S : Emotional Intelligence A Literature Review. 2007 Jan 1.

[87] Salovey P Mayer JD Caruso D : The positive psychology of emotional intelligence. *Handbook of positive psychology.* New York, NY, US: Oxford University Press;2002; pp.159–171.

