# Supplementary material for: Emotional intelligence: An important skill to learn now more than ever
Source: F1000Res. 2023 Sep 13;12:1146. [Version 1] doi: 10.12688/f1000research.134752.1 (PMC13305550; doi:10.12688/f1000research.134752.1)
Supplement: Peer review history

# Reviewer report: Reviewer response for version 1

**DOI:** 10.5256/f1000research.147832.r495241

**Published:** 2026-06-26

**Authors:** Smriti Agnihotri

**Affiliations:** 1 Health Sciences Education and Pathology, University of Illinois College of Medicine, Chicago, IL, USA

The article, “Emotional Intelligence: An Important Skill to Learn Now More Than Ever,” provides a clear and useful overview of emotional intelligence (EI). It explains the development of the concept of EI, introduces the major theoretical models, and describes the commonly used tools for measuring EI. The article discusses important contributors to the field, including Gardner, Thorndike, Mayer and Salovey, Goleman, and Bar-On. It also provides a helpful description of several EI assessment instruments, including the Multifactor Emotional Intelligence Scale, Mayer-Salovey-Caruso Emotional Intelligence Test, Self-Report Emotional Intelligence Test, Trait Emotional Intelligence Questionnaire, Bar-On Emotional Quotient Inventory, Situational Test of Emotional Management, Situational Test of Emotional Understanding, and Emotional and Social Competence Inventory.

 The topic is timely and relevant for readers in psychology, education, healthcare, and organizational settings. Emotional intelligence has increasing importance in understanding emotional regulation, interpersonal communication, teamwork, leadership, well-being, and professional development. The article successfully brings together the main concepts, theories, and measurement approaches in one place. This makes it a valuable introductory resource for students, educators, healthcare professionals, and others who wish to understand the foundations of emotional intelligence.

 The review is written in accessible language and follows a logical structure. The progression from the history and theories of intelligence to the models of EI and the assessment tools is easy to follow. The explanations of the major EI models are particularly useful because they help readers understand the differences between ability-based, trait-based, and mixed models of EI. The discussion of assessment instruments is also a strength of the article, as it provides readers with a broad understanding of the different ways EI can be evaluated.

 The article presents a balanced overview of both the usefulness and the ongoing development of EI research. It recognizes that EI is related to important outcomes such as emotional well-being, interpersonal functioning, workplace performance, and adaptation to stressful situations. The review also appropriately notes that EI is a complex construct and that different tools may measure different aspects of emotional functioning. This is an important contribution because it helps readers appreciate that EI is not a single, simple skill but a broad area that includes emotional perception, understanding, regulation, empathy, and social competence.

 The article would be further strengthened by adding a small number of more recent studies and reviews to demonstrate how EI research continues to develop in current healthcare, educational, workplace, and mental-health settings. Including recent evidence would enhance the contemporary relevance of the review while maintaining its strong foundation in the classic literature. A concise comparison table of the main EI models and assessment tools could also be helpful for readers, especially students and professionals who are new to the field.

 Overall, this is a well-organized and informative review of emotional intelligence. The article provides a useful summary of the history, major theories, models, and measurement tools related to EI. Its accessible language and broad scope make it suitable for a wide readership. With minor revisions to include a few recent references and further enhance clarity in selected sections, the article would be a valuable resource for readers interested in emotional intelligence and its applications in personal, educational, healthcare, and professional settings. I support indexing after minor revision.

Is the review written in accessible language?

Yes

Are all factual statements correct and adequately supported by citations?

Yes

Are the conclusions drawn appropriate in the context of the current research literature?

Yes

Is the topic of the review discussed comprehensively in the context of the current literature?

Yes

Reviewer Expertise:

Medical education

I confirm that I have read this submission and believe that I have an appropriate level of expertise to confirm that it is of an acceptable scientific standard.

# Reviewer report: Reviewer response for version 1

**DOI:** 10.5256/f1000research.147832.r495243

**Published:** 2026-06-24

**Authors:** Sumit Sontakke

**Affiliations:** 1 University of Mississippi Medical Center, Jackson, Mississippi, USA

Summary

 This review article provides a concise and informative overview of emotional intelligence (EI), including its historical development, major theoretical models, and commonly used assessment instruments. The manuscript successfully summarizes key concepts related to EI and discusses its potential relevance to mental health, physical health, and overall well-being. The topic is timely and of broad interest to researchers, educators, healthcare professionals, and students.

 Comments

 The manuscript is well organized and easy to follow. The authors provide a useful introduction to the evolution of emotional intelligence and present a balanced overview of several important EI models and measurement tools. The discussion of commonly used assessment instruments is particularly valuable for readers who are new to the field.

 The article serves as a helpful educational resource by bringing together information from multiple sources into a single, accessible review. The writing is clear, the scope is appropriate for a narrative review, and the conclusions are supported by the literature presented.

 The review highlights the potential importance of emotional intelligence in promoting emotional health and general well-being and provides readers with a solid foundation for further exploration of the topic.

 Recommendation

 I enjoyed reading this manuscript and believe it provides a useful overview of emotional intelligence and its assessment. The article is informative, well written, and suitable for indexing in its current form.

Is the review written in accessible language?

Yes

Are all factual statements correct and adequately supported by citations?

Yes

Are the conclusions drawn appropriate in the context of the current research literature?

Yes

Is the topic of the review discussed comprehensively in the context of the current literature?

Yes

Reviewer Expertise:

Medical Education, Healthcare Simulation, Clinical Skills Assessment, Standardized Patients, Educational Assessment, Emotional Intelligence, Professionalism, Communication Skills, Learner Development and Well-being, Clinical Microbiology, Infectious Diseases, and Laboratory Medicine.

I confirm that I have read this submission and believe that I have an appropriate level of expertise to confirm that it is of an acceptable scientific standard.
